# The effect of Bushen Culuan Decoction on anovulatory infertile women among 6 different diseases: a study protocol for a randomized, double-blinded, positively controlled, adaptive multicenter clinical trial

**DOI:** 10.1186/s13063-022-06289-7

**Published:** 2022-07-08

**Authors:** Kun Ma, Yun Shi, Junqin He, Xiuxiang Teng, Rongyu Wang, Guohua Wang, Yanan Yu, Yanxia Chen, Linjuan Gong, Yuan Yuan, Huixian Zhang, Bochao Yuan, Chenhui Zhang

**Affiliations:** 1 Xiyuan Hospital, China Academy of Chinese Medical Science, Beijing, 100091 China; 2 Dongzhimen Hospital, Beijing University of Chinese Medicine, Beijing, 100700 China; 3 Beijing Obstetrics and Gynecology Hospital, Capital Medical University, Beijing Maternal and Child Health Care Hospital, Beijing, 100006 China; 4 Beijing Hospital of Traditional Chinese Medicine, Beijing, 100010 China; 5 Beijing First Hospital of Integrated Chinese and Western Medicine, Beijing, 100026 China; 6 Beijing University of Chinese Medicine Third Affiliated Hospital, Beijing, 100029 China; 7 Institute of Basic Research in Clinical Medicine, China Academy of Chinese Medical Sciences, Beijing, 100007 China

**Keywords:** Anovulatory infertility, Basket design, Randomized controlled trial

## Abstract

**Background:**

Anovulation is one of the main causes of female infertility. This study will evaluate the effectiveness and safety of Bushen Culuan Decoction for anovulatory infertility caused by six diseases, including anovulatory abnormal uterine bleeding, polycystic ovarian syndrome, hyperprolactinemia, luteinized unruptured follicle syndrome, corpus luteum insufficiency, and premature ovarian insufficiency.

**Methods:**

This is a randomized, double-blinded, double-dummy, parallel, positively controlled, adaptive, multicenter clinical trial. All participants will be randomly allocated by a central randomization system to the treatment group or the control group in a 1:1 ratio. The treatment group will undergo a 14-day treatment with Bushen Culuan Decoction 13 g three times a day and a 5-day treatment with clomiphene citrate placebo tablets 50 mg once a day starting on day 5 of every menstrual period. The control group will undergo a 14-day treatment with Bushen Culuan Decoction placebo 13 g three times a day and a 5-day treatment with clomiphene citrate tablets 50 mg once a day from day 5 in every menstrual period. The whole treatment will last through 3 menstrual periods or 6 menstrual periods, depending on whether ovulation is regained in the first 3 menstrual periods. All statistical analyses will be performed in SPSS 21.0 (SPSS, Chicago, Illinois, USA), and a *p* value < 0.05 will be considered statistically significant.

**Discussion:**

The objective of this RCT is to evaluate whether Bushen Culuan Decoction enables a higher pregnancy rate than clomiphene citrate in women with anovulatory infertility and to identify the anovulatory diseases for which Bushen Culuan Decoction has higher effectiveness .This study has been approved by the Medical Ethics Committee of Xiyuan Hospital China Academy of Chinese Medical Sciences (No. 2017XLA037-2). The results of this study will be offered for publication in peer-reviewed journals.

**Trial registration:**

ClinicalTrials.gov NCT03709849. Registered on 19 November 2018.

## Background

Anovulation is one of the main causes of female infertility, contributing to 25–30% of all female infertility [1]. The function of the hypothalamic-pituitary-ovarian axis (HPOA) can be disturbed by psychentonia, over-fatigue, and diseases related to the hypothalamus, pituitary, ovary, thyroid or adrenal gland, and malfunction of the HPOA will lead to follicle hypoplasia, delayed follicle maturation, or anovulation [2]. Depending on the pathological mechanisms, the causes of anovulatory infertility can be divided into several categories, including abnormal uterine bleeding-ovulatory disorders (AUB-O), polycystic ovarian syndrome (PCOS), hyperprolactinemia, luteinized unruptured follicle syndrome (LUFS), corpus luteum insufficiency, and premature ovarian insufficiency (POI).

In Western medicine, hormone-induced ovulation and assisted reproduction technology are two main ways to treat anovulatory infertility. The commonly used drugs include clomiphene citrate, letrozole, gonadotropins (Gn), and gonadotropin-releasing hormone (GnRH). ART contains intrauterine insemination (IUI) with or without controlled ovarian hyperstimulation (COH), in vitro fertilization-embryo transfer (IVF-ET), gamete intrafallopian transfer (GIFT), oocyte donation, intracytoplasmic sperm injection (ICSI), preimplantation genetic diagnosis (PGD), etc. [3]. There are several side effects that need to be considered during treatment using modern drugs or techniques. (1) Ovarian hyperstimulation syndrome (OHSS): the overall morbidity of OHSS in ovulation induction is 1–14%. The serious morbidity rate of OHSS is 0.5–2%, including tense ascites, liver and renal function damage, adult respiratory distress (ARDS), and thromboembolism [4]. (2) Complications of puncture and peritoneoscopy when collecting oocytes: these include puncture injury and bleeding, complications of anesthesia, pneumothorax, and mediastinal emphysema in peritoneoscopy and thrombus [5]. (3) Ovulation induction may bring a risk of a high ovulation rate with a low pregnancy rate, high miscarriage rate, multiple pregnancy, and ectopic pregnancy. (4) Ovulation induction surpassing the natural period could elevate the risk of cancer, such as breast cancer, cancer of the genital tract, and melanoma [6–8].

Current clinical trials have shown that traditional Chinese medicine (TCM) was associated with significantly higher pregnancy rates than control treatments, and the ovulation rates between the two groups were not significantly different [9–11]. It is notable that all systemic reviews and meta-analyses have mentioned that there is a lack of high-quality, strong, evidence-based, large-sample randomized controlled trials (RCTs) on this topic.

Bushen Culuan Decoction was created more than 20 years ago. In a previous experimental study evaluating the effectiveness and safety of Bushen Culuan Decoction in treating anovulatory infertility, it was found that Bushen Culuan Decoction had no adverse effect or toxic reaction in acute toxicity tests, reproductive toxicity tests, genetic tests, and teratogenic toxicity tests [12] and significantly promoted follicle maturation, ovulation, and luteinization [13]. A clinical exploratory study showed that Bushen Culuan Decoction significantly increased the pregnancy rate, depressed serum prolactin (PRL), and elevated serum estradiol (E_2_) compared with the control treatment in women with anovulatory infertility [14].

Until now, most studies about TCM treating anovulatory infertility have focused on a single disease in a single center, and there is no study with a large sample size aiming to verify the effectiveness and safety of Bushen Culuan Decoction in treating anovulatory infertility. Based on the results of previous studies, we hypothesized that Bushen Culuan Decoction would improve the pregnancy rate by promoting follicle development and ovulation and improving endometrial receptivity. This study will evaluate that hypothesis.

### Objectives

The objective of this RCT is to evaluate whether Bushen Culuan Decoction enables a higher pregnancy rate than clomiphene citrate in women with anovulatory infertility and to identify the anovulatory diseases for which Bushen Culuan Decoction has higher effectiveness (among AUB-O, PCOS, hyperprolactinemia, LUFS, corpus luteum insufficiency, and POI).

## Methods

### Design

**Fig. 1:**
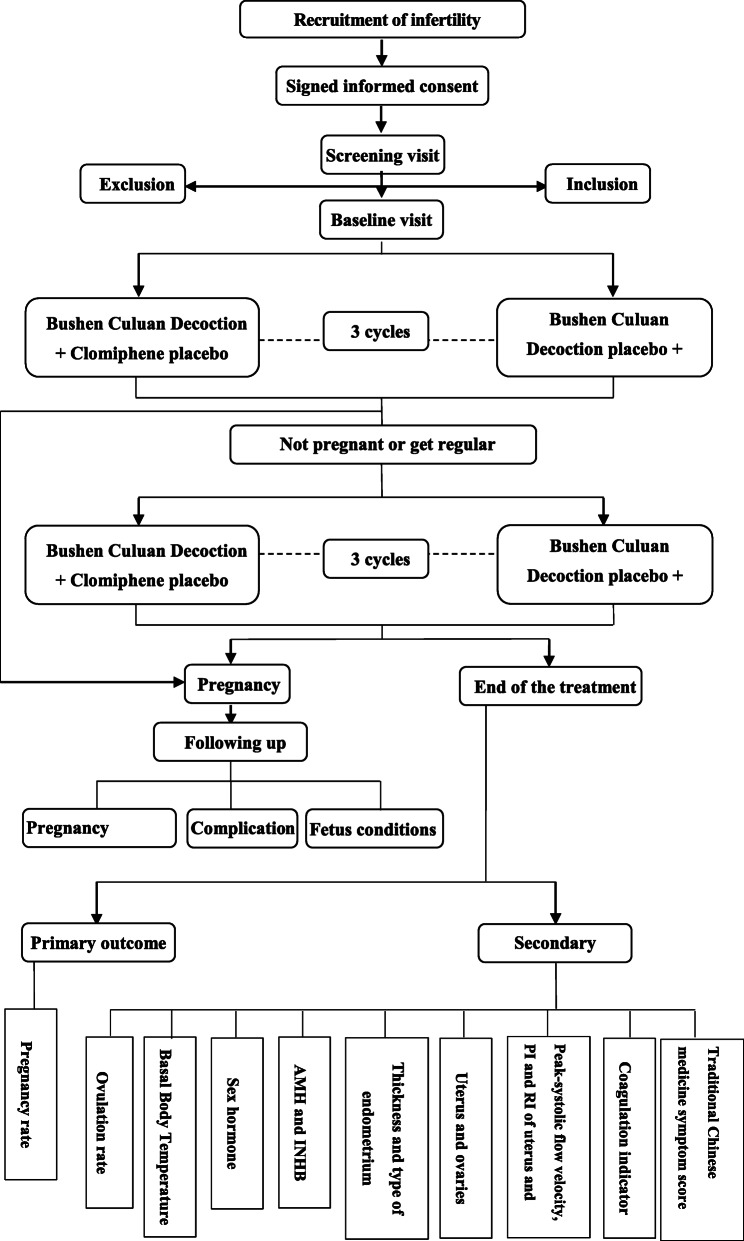
Study flow chart. AMH: anti-Mullerian hormone; INHB: inhibin B; PI: pulsatility index

**Fig. 2:**
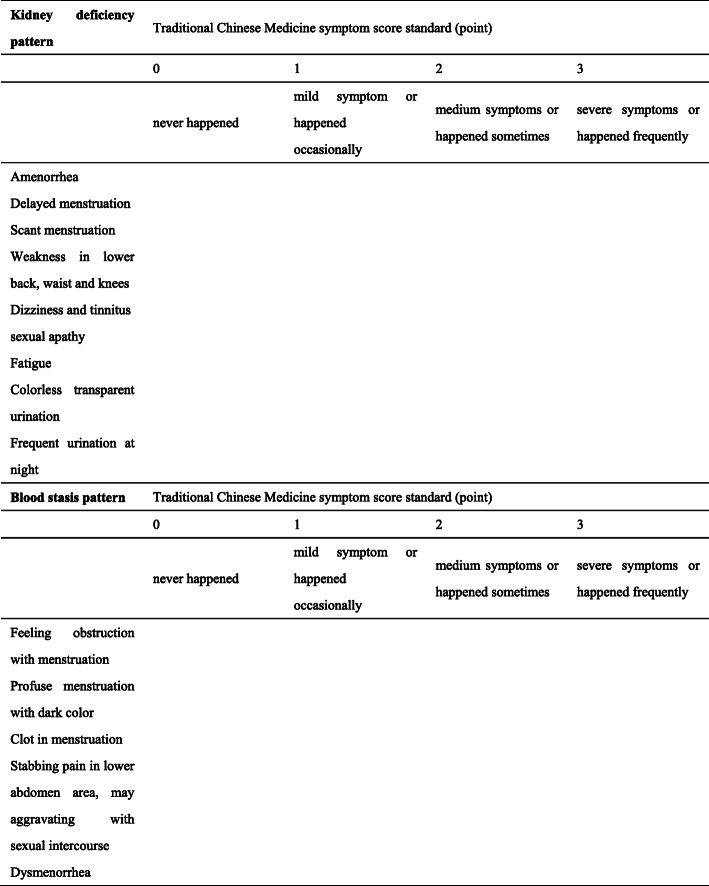
Traditional Chinese Medicine symptom score

This is a randomized, double-blinded, double-dummy, parallel, positively controlled, adaptive, multicenter clinical trial comparing the pregnancy rate after treatment with Bushen Culuan Decoction versus treatment with clomiphene citrate in women suffering from anovulatory infertility caused by AUB-O, PCOS, hyperprolactinemia, LUFS, corpus luteum insufficiency, or POI, and the flow chart and assessment timepoints are shown in Figs. 1 and 2. The study protocol will be drafted in accordance with the guidelines of the Standard Protocol Items: Recommendations for Interventional Trials (SPIRIT). This study has been registered at www.clinicaltrail.gov (NCT03709849).

### Patient and public involvement

At the stage of research scheme formulation, the research group invited patients to participate in the scheme feasibility review meeting, and the researchers fully listened to and adopted the opinions of patients, especially in determining the return visit time and drafting informed consent. The final report of the patient’s study will also be fed back to the patient.

### Ethics approval

This study has been approved by the Medical Ethics Committee of Xiyuan Hospital China Academy of Chinese Medical Sciences (No. 2017XLA037-2). Ethical amendment approval will be required if the protocol needs to be modified. All patients are required to sign an informed consent form before joining the study. Clinical investigators must make sure that the participant knows very well the whole process of the study and the benefits and risks she will be subject to whether she is in the experimental group or the control group.

### Sample size estimation

In a previous study, the total effective rate (Total effective rate% = (markedly effective + effective) / number of patients × 100%) in the experimental group was 90%, and the total effective rate in the control group was 80%. Thus, the difference between the two groups was 10%. We used the calculation formula for superiority trials to calculate the sample size, and the *α* error and *β* error were set at 0.05 and 0.10, respectively. This will require a sample size of 219 participants in each group. The dropout rate is expected to be 20%, which means that a total of 528 patients are needed for the two groups.

This study is an adaptive research study with an interim analysis. In the first stage of the study, 6 hospitals will screen 1/3 of the total sample size, which will be 176 patients. When we calculate the pregnancy rate, the primary outcome of this study, we will go through the first unblinding and interim analysis. The aim of the interim is to find out which of the six diseases are more sensitive to Bushen Culuan Decoction therapy. If Bushen Culuan Decoction shows significant clinical benefit or tends toward a benefit for a certain disease, research on this disease will go into the second stage of the study. If Bushen Culuan Decoction does not reach the treatment effectiveness threshold, research on this disease will be suspended in the next stage. At this time, the sample size will be recalculated according to the interim analysis result.

### Participants

All participants will be evenly recruited from six hospitals in Beijing, China: Xiyuan Hospital of China Academy of Chinese Medical Sciences, Dongzhimen Hospital, Beijing Obstetrics and Gynecology Hospital, Beijing Hospital of Traditional Chinese Medicine, Beijing First Hospital of Integrated Chinese and Western Medicine and Beijing University of Chinese Medicine Third Affiliated Hospital.88 participants will be recruited from every hospital. In order to let more patients see our recruitment, we will publicize the official account and website of WeChat, and put up posters in each subcenter.

#### Inclusion criteria

Participants are women aged 21–40 years old who have been diagnosed with infertility and one of the following diseases: anovulatory abnormal uterine bleeding, polycystic ovarian syndrome, hyperprolactinemia, luteinized unruptured follicle syndrome, corpus luteum insufficiency and ovarian insufficiency; who have been diagnosed with the TCM syndrome of kidney deficiency pattern and blood stasis pattern; who have regular sexual intercourse during treatment; and who voluntarily sign the informed consent.

#### Exclusion criteria

The study excludes participants who have infertility due to congenital reproductive system physiological defects or malformations; hereditary factors; oviduct defects; immune factors; uterine fibroids; adenomyosis or endometriosis; spouses with reproductive defects; severe abnormalities of the cardiovascular system, liver function, kidney function or hemopoietic system; or allergies to experimental drugs.

#### Discontinuation criteria

Treatment will be discontinued when the PI deems the trial harmful for the participant.

The whole trial will be terminated if the clinical trial is canceled by authorities or serious adverse events happen during the trial.

### Withdrawal criteria

Participant have the right to withdraw from the study, as defined in the informed consent document, or to “drop out” if they do not explicitly withdraw from the study but are no longer undergoing training and testing. The reason for withdrawal should be understood as far as possible and recorded, for example, perceiving lack of efficacy; intolerance of certain adverse effects; inability to continue the clinical study; and lost to follow-up without explanation. Case record forms for withdrawn participant will be kept, and the results of the last trial will be considered final.

### Diagnostic criteria of related diseases

Infertility is the failure to establish a clinical pregnancy after 12 months of regular, unprotected sexual intercourse, either primary or secondary [15].

#### Abnormal uterine bleeding-ovulatory disorders (AUB-O)

1. Uterine bleeding has no cyclicity or regularity, and the bleeding amount cannot be estimated. Anemia or hemorrhagic shock will mark severe cases.
2. Excludes bleeding caused by pregnancy or pregnancy-related factors, bleeding after menopause, bleeding from the genital tract except the uterus or from nongenital organs, iatrogenic bleeding, and bleeding caused by systemic disease.
3. Basal body temperature (BBT) appears as a single phase.
4. One or more sex hormone levels are beyond the normal range, including follicle-stimulation hormone (FSH), luteinizing hormone (LH), prolactin (PRL), estradiol (E_2_), progesterone (P), and testosterone (T).

AUB-O will be diagnosed if both (1) and (2) are met and at least one of (3) and (4) is met [16].

#### Polycystic ovarian syndrome (PCOS)

1. Oligomenorrhea or amenorrhea: oligomenorrhea means a menstrual period > 35 days or < 8 cycles in the past 2–3 years after menarche, and amenorrhea means a duration of the menstrual period > 90 days. BBT shows a single phase.
2. Clinical and/or biochemical hyperandrogenism. The features of clinical hyperandrogenism include crinosity and acne, and biochemical hyperandrogenism is defined as total testosterone (T) > 2.6 nmol/L and free testosterone ≥ 6.0 pg/mL.
3. Polycystic ovarian morphology: Transvaginal ultrasound shows the presence of ≥ 12 antral follicles with follicle diameters ≥ 9 mm and/or ovarian volumes > 10 mL.

PCOS can be diagnosed if at least 2 of the 3 conditions are met [17].

#### Hyperprolactinemia

1. A fasting blood sample must be obtained between 9 and 11 a.m. when the patient is awake and calm. If the serum prolactin level is equal to or greater than 3 times the upper normal limit, which is 90 ng/ml, the blood test can be done only once. If the serum prolactin level is between 30 and 90 ng/ml, a second blood test is necessary. When the second serum prolactin level is still between 30 and 90 ng/ml, hyperprolactinemia can be diagnosed.
2. Clinical manifestation: oligomenorrhea, amenorrhea, galactorrhea, infertility, etc.

Hyperprolactinemia can be diagnosed if (1) is met [18].

#### Luteinized unruptured follicle syndrome (LUFS)

1. Regular menstruation with double-phased BBT.
2. Daily transvaginal ultrasound shows one of the following:
  - Small follicle luteinization type: follicle volume does not change on the estimated ovulation day, with the intrafollicular spot fading away;
  - Follicle-retention type: follicle volume remains at 25 mm in diameter on the estimated ovulation day, and the wall of the follicle cyst thickens gradually. Strong echogenic dots appear 2–4 days from the estimated ovulation day and then fade away gradually.
  - Follicle continuous enlarging type: the diameter of the follicle is 31–50 mm on the estimated ovulation day, without free fluid in the Douglas pouch.
3. Fasting blood sample for assaying sex hormones shows that serum progesterone at the mid-luteal phase reaches the postovulation range.
4. Qualified BBT, ultrasound image, and blood test result continue for at least two menstrual periods.

LUFS can be diagnosed if both (2) and (4) are met and at least one of (1) and (3) is met [19].

#### Corpus luteum insufficiency

1. Clinical features: The menstrual period is less than 21 days, or the menstrual phase is longer than 7 days with vaginal spotting before menstruation. Patients may have a history of early spontaneous abortion.
2. BBT shows that the duration of the luteal phase is less than 11 days, or the descent of BBT from the high-temperature phase is flat.
3. Fasting blood samples for assaying sex hormones taken 5–9 days before menstruation show that the serum progesterone level is lower than the normal range.

Corpus luteum insufficiency can be diagnosed if (1) is met and at least one of (2) and (3) is met [20].

#### Premature ovarian insufficiency

1. Oligo-/amenorrhea for at least 4 months.
2. FSH > 25 IU/I on two occasions > 4 weeks apart.

POI can be diagnosed if (1) and (2) are met [21].

### Blinding

A central randomization system and random envelopes will be used to allocate patients. We will use a two-grade, double-blind, double-dummy design, and the first-grade blinding will ensure that all experimental drugs and control drugs have the same external packing so that both primary investigators and participants will not know which one the participant takes until the unblinding stage. The second-grade blinding refers to the random number and blinding code.

### Randomization

Using the stratified block randomization method, a special researcher at the Institute of Basic Research in Clinical Medicine, China Academy of Chinese Medical Sciences (IBRCM, CACMS) is responsible for compiling the number sequence table in turn, generating random numbers through SAS 9.3statistical software, determining them in the form of documents after coding, and keeping them by special personnel after sealing. According to the grouping results obtained, the clinical investigator gives the corresponding drugs to the patient according to the subject sequence number and drug number.

### Allocation concealment

The blind codes will be sealed and kept secure at IBRCM. The first-grade unblinding will be done before data analysis, and the second-grade unblinding will be done after we calculate the statistical results.

### Interventions

Eligible patients will be randomly allocated into one of the two arms. Experimental group: Bushen Culuan Decoction, containing the ingredients of Ligustrum lucidum, dodder, Xianling spleen, Morinda officinalis, Eupatorium adenophorum, Salvia miltiorrhiza, Angelica sinensis, Ligusticum chuanxiong, red peony, raw dandelion (made by Beijing Tcmages Pharmaceutical Co, Beijing) with clomiphene citrate placebo tablets (made by the drug manufacturing department of Xiyuan Hospital). Both drugs will be started on day 5 after a spontaneous menstrual period or after vaginal withdrawal bleeding following progesterone (100 mg twice a day for 6 days, Zhejiang Xianju Pharmaceutical Co, Taizhou) if the participant does not have a regular menstrual period. Bushen Culuan Decoction will be taken at 13 g three times a day for 14 days, while clomiphene citrate placebo tablets will be taken at 50 mg once a day for 5 days. Each treatment cycle will contain 3 menstrual periods. If the participant regains regular ovulation at the end of the first, her treatment will end, and if not, she will start the second treatment cycle. All treatments will be terminated after 2 treatment cycles. If the participant is pregnant, all treatment will be stopped, and she will be moved into the follow-up stage.

Control group: clomiphene citrate tablets with Bushen Culuan Decoction placebo treatment (made by Beijing Tcmages Pharmaceutical Co, Beijing). Clomiphene citrate tablets (Fertilan®, Codal Synto Ltd. Cyprus) will be taken at 50 mg once a day for 5 days, while Bushen Culuan Decoction placebo will be taken at 13 g three times a day for 14 days. The starting time, termination time, and treatment cycle will be the same as those of the experimental group.

### Study-specific visits and procedures

The whole experiment for every participant will involve treatment with Bushen Culuan Decoction+ clomiphene citrate placebo or clomiphene citrate+ Bushen Culuan Decoction placebo for three menstrual periods or six menstrual periods (Fig. 1). Every participant will attend up to five visits, including the screening visit, baseline visit, treatment visit, end-of-treatment visit, and follow-up visit. The overview of the study visits is shown in Table 1. All concomitant medications and adverse events will be recorded at every visit.

**Table 1:** Overview of study visits

Overview of study visits								
	Screening visit	Baseline visit	Treatment visit				End-of-treatment visit	Follow-up visit
			1st and 2nd menstrual period	3rd menstrual period	4th and 5th menstrual period	6th menstrual period		
Physical examination	√							
Basal body temperature		√	√	√	√	√	√	
Fasting blood sample for safety profile		√					√	
Urine routine test for safety profile		√					√	
ECG for safety profile		√					√	
Pregnancy test*		√	√	√	√	√	√	√
Fasting blood sample for sex hormone steroids		√		√		√	√	
Transvaginal ultrasound for ovulation monitoring		√	√	√	√	√	√	
Blood sample for AMH and INHB		√					√	
Transvaginal ultrasound		√					√	
Coagulation indicator		√					√	
Traditional Chinese Medicine symptom score**		√	√	√	√		√	
Pregnancy and neonatal record								
Query for adverse events and concomitant medications		√	√	√	√	√	√	

Physical examination: weight and height for BMI; acne and hair observation

Fasting blood samples for safety profile: RBC, Hb, WBC, PLT, ALT, AST, BIL, BUN, Cr

Urine routine test for safety profile: WBC-M, RBC-M, PRO

Pregnancy test: will be tested if the high-temperature phase of BBT continues for more than 14 days in a menstrual period, or the menstrual period is longer than 28 days

^*^Degree of importance

Fasting blood samples for sex hormones: FSH, LH, PRL, E_2_, P, T

Transvaginal ultrasound: uterine and bilateral ovarian volumes, endometrial thickness and type, ovarian volume, antral follicle count, and sizes of ovarian cysts or developing follicles. Peak-systolic flow velocity, PI and RI of uterus and ovaries

Coagulation indicator: PT, TT, APTT, FIB

Traditional Chinese Medicine symptom score is shown in Table 2

*RBC* red blood cells, *Hb* hemoglobin, *WBC* white blood cells, *PLT* platelets, *ALT* glutamic-pyruvic transaminase, *AST* glutamic-oxalacetic transaminase, *BIL* bilirubin, *BUN* blood urea nitrogen, *Cr* creatinine, *RBC-M* red blood cells (high-power field), *WBC-M* white blood cells (high-power field), *PRO* protein, *FSH* follicle-stimulating hormone, *LH* luteinizing hormone, *PRL* prolactin, *E*_*2*_ estradiol, *P* progesterone, *T* testosterone, *PT* prothrombin time, *TT* thrombin time, *APTT* activated partial thromboplastin time, *FIB* fibrinogen *PI* pulsatility index, *RI* resistant index, *AMH* anti-Mullerian hormone, *INHB* inhibin B

*degree of importance

### Obtain signed informed consent

Every patient who agrees to participate in the study must sign the informed consent form (ICF). First, the clinical investigator will explain every detail relevant to the study to the potential participant, including the whole procedure; what the participant will do before, during, and after the treatment; the benefits and risks she will face; how to deal with any possible adverse events; making sure the participant fully understands the study. Every participant will get one copy of the ICF after signing it, and then she can move into the screening stage.

### Screening visit

Every participant who is willing to join in the study will have a face-to-face consultation with one of the clinical investigators to verify that they meet the basic inclusion criteria, such as age, reproductive history, and sexual intercourse condition. Those who are eligible will be scheduled for a screening visit.

Every participant will get a basal body temperature (BBT) form once she has joined the study. The BBT will be monitored and recorded on the form by the participant herself every morning from the day after the screening visit, and the result will be recorded in the CRF at every visit from the baseline visit.

### General history

Birth date (yyyy/mm/dd), height (to the nearest 0.1 cm), weight (to the nearest 0.1 kg), whether the participant has a physical job, and whether she is a smoker will be recorded.

### Fertility history

We will record the participant’s information, including the duration of infertility with normal intercourse, the number of pregnancies, the number of spontaneous abortions, the number of artificial abortions, and embryo damage.

### Menstrual history

The age of menarche, the durations of the menstrual period and menstruation, and the date of last menstruation period (LMP) of the participant will be recorded. We will also obtain information about the relevant treatment, including the treatment duration and medication she used.

### Diagnosis of the 6 diseases

The participant must have a diagnosis of one of the 6 diseases based on the diagnostic criteria mentioned above.

### Exclusion of other infertility factors of the couple

Every participant will be confirmed to have no congenital reproductive system malformation, uterine fibroid, adenomyosis, or endometriosis. The tubal patency and the semen quality of her partner will be confirmed to be satisfactory to get pregnant. Both the woman and her partner must have normal results of routine chromosome screening.

### Baseline visit

Each participant who is eligible for the study as screened in the last step will be scheduled for a baseline visit. We will collect general vital signs, safety index, and effectiveness index before the treatment in this stage.

### General vital signs

The general vital signs collected will include body temperature, pulse, respiratory frequency, and blood pressure (systolic blood pressure/diastolic blood pressure).

### Safety index

Fasting blood samples will be collected for testing routine blood tests, including RBC, Hb, WBC, and PLT. Liver and renal function tests will include ALT, AST, BIL, BUN, and Cr. A urine sample will be collected for WBC-M, RBC-M, and PRO. An ECG will be taken for routine heart function.

### Effectiveness index

The effectiveness index is divided into 6 parts.

1. The date and duration of LMP and BBT analysis. BBT will be analyzed by the clinical investigator, and the result will be categorized as single-phase, double-phase, or atypical double-phase, along with the duration of the high-temperature phase.
2. Sex hormone. Sex hormones, including FSH, LH, PRL, E2, P, and T, will be taken one or two times before the baseline visit and will be recorded at the baseline visit. The first blood sample collection will be in the follicular phase of the menstrual period, which is days 3–5 of the menstrual period. The second blood sample collection will be at the ovulatory phase, as confirmed by a positive ovulation test result. If the participant does not have a typical menstrual period or cannot identify the ovulatory phase, she will take the blood sample once and record the date of LMP.
3. Serum INHB and AMH. These two indicators will not significantly change within a menstrual period, so we will take them at the same time as the first collection of sex hormones.
4. Coagulation indicators. Blood samples will be collected at the same time as the first collection of sex hormones in order to measure coagulation indicators, including PT, TT, APTT, and FIB.
5. Transvaginal ultrasound. The first transvaginal ultrasound will be done 3–5 days after the menstrual bleeding has ended. The information collected at this time will be uterine and bilateral ovarian volumes, the thickness and type of endometrium, bilateral ovarian antral follicle count, uterine and bilateral ovarian peak-systolic flow velocity, pulsatility index, and resistance index. On the 12th day of the menstrual period, the participant will start to monitor the ovulation every day, and the size of the dominant follicle will be recorded.
6. Traditional Chinese medicine symptom score. The clinical investigator will show the participant the form and explain every detail to ensure that the participant fully understands it and let her choose every answer. The form is shown in Table 2.

**Table 2:** Traditional Chinese Medicine symptom score

Kidney deficiency pattern	Traditional Chinese Medicine symptom score standard (point)			
	0	1	2	3
	Never happened	Mild symptom or happened occasionally	Medium symptoms or happened sometimes	Severe symptoms or happened frequently
Amenorrhea				
Delayed menstruation				
Scant menstruation				
Weakness in lower back, waist and knees				
Dizziness and tinnitus				
sexual apathy				
Fatigue				
Colorless transparent urination				
Frequent urination at night				
Blood stasis pattern	Traditional Chinese Medicine symptom score standard (point)			
	0	1	2	3
	Never happened	Mild symptom or happened occasionally	Medium symptoms or happened sometimes	Severe symptoms or happened frequently
Feeling obstruction with menstruation				
Profuse menstruation with dark color				
Clot in menstruation				
Stabbing pain in lower abdomen area, may aggravating with sexual intercourse				
Dysmenorrhea				

After all the above information is recorded, the participant will be given a package of medication. The methods of taking the medicines are shown on the pack and will be explained by the clinical investigator. The clinical investigator will also instruct the participant on how to have effective intercourse during the ovulatory phase and how to deal with any possible problems she may face, then make an appointment for the next visit.

### Treatment visit

After getting the medication package, every participant will start medical treatment on day 5 of the 1st menstrual period and visit the clinical investigator on day 25 of the 1st menstrual period or on day 1 of the 2nd menstrual period if the menstrual period duration is less than 25 days. In every treatment visit, relevant information about the participant will be recorded, and she will be given the medication package for the next menstrual treatment period.

In the 1st and 2nd menstrual periods, the participant will start to monitor ovulation every day from the 12th day of menstruation, and the size of the dominant follicle will be recorded. If there is ovulation, a urine pregnancy test will be performed to exclude pregnancy. If there is no pregnancy, the treatment will be continued in the next period. The detailed medication use status, the date and duration of the last menstruation period along with BBT, and ovulation monitoring results will be recorded, and the traditional Chinese medicine symptom score will be evaluated.

In the 3rd menstrual period, serum sex hormones at the ovulatory phase will be tested and recorded in addition to all information collected in the previous period. If there is no ovulation, the blood sample for sex hormone testing will be collected on the 15th day of the menstrual period.

After treatment for 3 periods, we will analyze the ovulation of every participant. The participant will be considered to have regained regular ovulation successfully if she had ovulation in all of the past 3 periods. The treatment of the participant will be terminated, and she will have an end-of-treatment visit and then move forward into the follow-up stage. If the participant ovulates fewer than 3 times, she will go into the second treatment cycle.

For every participant in the second treatment cycle, she will have a 4th, 5th, and 6th treatment visit. The procedures of the 4th and 5th treatment visits will be the same as those of the 1st and 2nd visits, and the 6th visit will be the same as that of the 3rd visit. After the 6th menstrual period treatment, all participants will stop treatment and move into the next stage regardless of whether they recover regular ovulation.

If the participant gets pregnant in the treatment process, she will stop the treatment immediately and move into the end-of-treatment visit and follow-up stage.

### End-of-treatment visit

The information to be collected and the procedure will be the same as in the baseline visit, including general vital signs, safety index, and effectiveness index. If the participant is pregnant, the transvaginal ultrasound will be skipped. The clinical investigator will inform every unpregnant participant that she will be contacted by phone every 3 months to have a follow-up visit until she is pregnant or until 12 months after the end of treatment. If the participant is pregnant in the treatment or follow-up stage, phone follow-up will be continued until there is a pregnancy outcome.

### Follow-up visit

After the end-of-treatment visit, participants who are not pregnant will come to follow-up visits every 3 months. In each visit, we will record the date and duration of the last menstrual period, her pregnancy situation, and a simple questionnaire for traditional Chinese medicine symptoms. For participants who are pregnant already, we will keep in touch with them until there is a pregnancy outcome.

The pregnancy outcomes are divided into

1. Natural labor;
2. Cesarean section;
3. Biochemical pregnancy;
4. Spontaneous abortion;
5. Artificial abortion.

For (1) and (2), we will record the weight, height, head circumference, and Apgar score of the newborn. For (4) and (5), the precipitating factors and relevant medical report are required to be offered by the participant.

### Outcome measures

#### Primary outcome

The primary outcome is pregnancy rate (confirmed by serum β-HCG positivity).

#### Secondary outcomes

Secondary outcomes are as follows:

1. Ovulation rate;
2. Basal body temperature;
3. Serum sex hormones: FSH, LH, PRL, E_2_, P, T;
4. Serum AMH and inhibin B;
5. Uterine and bilateral ovarian volumes at the early follicle phase;
6. Thickness and type of endometrium at the early follicle phase;
7. Antral follicle count and the size of dominant follicle;
8. Peak-systolic flow velocity, pulsatility index, and resistance index of the uterus and bilateral ovaries;
9. Serum coagulation indicators;
10. Traditional Chinese medicine symptom score.

### Safety analysis

Safety will be analyzed by adverse events (AEs); laboratory tests, including routine blood and urine tests, liver and renal function tests; and ECG. AEs will be recorded at every visit, and the other tests will be done at the baseline visit and end-of-treatment visit. Every AE will be recorded in detail, including the starting time and ending time, severity, characteristics (continuous or paroxysmal, and the frequency), any relationship with the drugs used in the study, the outcome, use or nonuse of corrective treatment, and whether the woman drops out of the study because of the AE. In this study, common AEs will include nausea, loose stool with dark color, and mild pain in the lateral lower abdomen on ovulation day, which are not expected to be severe and have no need of intervention. If the participant has serious adverse events (SAEs) leading to extended hospitalization, death, or a status that the PI considers unsuitable for further study, she will be removed from the trial as soon as possible and provided proper treatment. Every SAE will be recorded in the CRF and reported to the ethics committee and related health administrative department.

The degrees of safety analysis are divided into 4 grades according to the AE and the safety index profile:

- Grade 1: The participant is safe without any AE. There is no abnormal result in the safety index.
- Grade 2: The participant is relatively safe with a mild AE. The adverse reaction has no need of treatment, and the participant can continue the study. There is no abnormal result in the safety index.
- Grade 3: There is moderate AE, or there is a mildly abnormal result in safety index. The participant can continue the study after corrective intervention for the AE.
- Grade 4: The participant should be removed due to the SAE, or there is a moderate or serious abnormal result in safety index.

### Data management and quality control of the data

All original data will be recorded in case report forms (CRFs). Two keyboard operators will type all data in the analysis system twice independently, and a verification of data consistency will be done. The data administrator will further examine the data by using a logical consistency check system to ensure the veracity of the data, and then the database will be locked for statistical analysis.

All clinical investigators and relevant staff will be trained in good clinical practice (GCP) and the study protocol before the study starts. After completing all training processes, they will receive authorization to perform the study.

#### Statistical analysis

An independent statistician will use SPSS 21.0 (SPSS, Chicago, Illinois, USA) to analyze the data.

The mean ± SD will be used to describe normally distributed data, while medians with IQRs will be used if the data are not normally distributed. The *χ*^2^ test will be used for evaluating differences between dichotomous variables, while one-way analysis of variance or Student’s *t* test will be used for evaluating continuous variables. The Mann-Whitney test will be used if the data are not normally distributed. A *p* value < 0.05 will be considered statistically significant.

If there are dropouts from the study, the last-observation-carried-forward principle will be used to compensate for the loss of data.

### Trial status

We are currently recruiting participants for this trial. The protocol was registered on ClinicalTrials.gov on 19 December 2018 with the identifier NCT03709849 and the unique protocol ID Z171100001017104. The date recruitment began was November 17, 2019, and the approximate date when recruitment will be completed will be February 20, 2022.

## Data Availability

The result of this study will be disseminated via peer-reviewed publications and conference presentation. Al the data will be available upon request.
